# Olaparib treatment for platinum-sensitive relapsed ovarian cancer by BRCA mutation and homologous recombination deficiency status Phase II LIGHT study primary analysis

**DOI:** 10.1016/j.ygyno.2022.06.017

**Published:** 2022-07-05

**Authors:** Karen Cadoo, Fiona Simpkins, Cara Mathews, Ying L. Liu, Diane Provencher, Colleen McCormick, Adam C. ElNaggar, Alon D. Altman, Lucy Gilbert, Destin Black, Nashwa Kabil, James Bennett, Jiefen Munley, Carol Aghajanian

**Affiliations:** a Memorial Sloan Kettering Cancer Center, and Weill Cornell Medical College, New York, NY, USA; b Department of Obstetrics and Gynecology, Jordan Center for Gynecologic Oncology at the Abramson Cancer Center, University of Pennsylvania, Philadelphia, PA, USA; c Program in Women’s Oncology, Department of Obstetrics and Gynecology, Women and Infants Hospital, Brown University, Providence, RI, USA; d CHUM Gynecologie-Oncologie, Montreal, QC, Canada; e Legacy Medical Group Gynecologic Oncology, Portland, OR, USA; f West Cancer Center and Research Institute, Memphis, TN, USA; g CancerCare Manitoba, Research Institute of Oncology and Hematology, University of Manitoba, Winnipeg Manitoba, Canada; h Division of Gynecologic Oncology, McGill University Health Centre, Montreal, Quebec, Canada; i Willis-Knighton Cancer Center, Shreveport, LA, USA; j AstraZeneca, Gaithersburg, MD, USA; k AstraZeneca, Cambridge, UK; l AstraZeneca, Wilmington, DE, USA

**Keywords:** Ovarian cancer, Olaparib, Treatment, Homologous recombination deficiency, BRCA

## Abstract

**Objective.:**

Olaparib treatment resulted in significant improvement in objective response rates (ORRs) and progression-free survival (PFS) over non-platinum chemotherapy in patients with *BRCA1/BRCA2*-mutated (BRCAm) platinum-sensitive relapsed ovarian cancer (PSROC) and ≥2 prior lines of platinum-based chemotherapy in the phase III SOLO3 study. LIGHT (NCT02983799) prospectively evaluated olaparib treatment for patients with PSROC and known BRCAm and homologous recombination deficiency (HRD) status.

**Methods.:**

In this phase II open-label multicenter study, patients with PSROC and ≥1 prior line of platinum-based chemotherapy were assigned to cohorts by presence of germline BRCAm (gBRCAm), somatic BRCAm (sBRCAm), HRD-positive tumors without BRCAm, or HRD-negative tumors. The primary endpoint was investigator-assessed ORR. Secondary endpoints included disease control rate (DCR) and PFS. Tumors were analyzed using Myriad BRACAnalysis CDx and myChoice HRD assays; HRD-positive tumors were defined using a genomic instability score of ≥42.

**Results.:**

Of 272 enrolled patients, 271 received olaparib and 270 were included in efficacy analyses. At data cut-off, ORRs in the gBRCAm, sBRCAm, HRD-positive, and HRD-negative cohorts were 69.3%, 64.0%, 29.4%, and 10.1%, respectively. DCRs were 96.0%, 100.0%, 79.4%, and 75.3% in each cohort, respectively. Median PFS was 11.0, 10.8, 7.2, and 5.4 months, respectively. The most common (≥ 20%) treatment-emergent adverse events included nausea, fatigue/asthenia, vomiting, anemia, constipation, diarrhea, and decreased appetite.

**Conclusions.:**

Olaparib treatment demonstrated activity across all cohorts. The greatest efficacy was observed in the BRCAm cohorts, regardless of gBRCAm/sBRCAm. For patients without a BRCAm, greater efficacy was observed in the HRD-positive than the HRD-negative cohorts. The safety profile was consistent with that established in previous olaparib studies.

## 1. Introduction

Although treatment of newly diagnosed ovarian cancer with debulking surgery and platinum-based chemotherapy initially results in high response rates, approximately 70% of patients will experience relapse within 3 years [1]. Recurrent ovarian cancer is unfortunately often incurable, and overall, ovarian cancer represents the eighth most common cause of cancer-related mortality among women worldwide [2].

Therapeutic advances, including poly(ADP-ribose)polymerase (PARP) inhibitors such as olaparib, have greatly improved patient outcomes. Maintenance olaparib significantly improved progression-free survival (PFS) in patients with platinum-sensitive relapsed ovarian cancer (PSROC) unselected for *BRCA1* and/or *BRCA2* mutation (BRCAm) status [3], and further in patients with a BRCAm who were in response to prior platinum-based chemotherapy [4], resulting in approval as maintenance therapy for patients with PSROC irrespective of BRCAm status [5–7]. In the newly diagnosed advanced setting, maintenance olaparib significantly improved PFS over placebo in patients with a BRCAm [8], and maintenance olaparib plus bevacizumab demonstrated a significant PFS improvement over placebo plus bevacizumab, particularly for patients with homologous recombination deficiency (HRD)-positive tumors (defined as a genomic instability score ≥ 42 and/or a BRCAm) [9].

Outside of the maintenance setting, olaparib was FDA-approved for the treatment of advanced ovarian cancer in patients with a germline (g) BRCAm who have received three or more prior lines of chemotherapy [5]. In the phase III SOLO3 trial, which included patients with a gBRCAm and ≥2 prior lines of platinum-based chemotherapy and randomized patients to receive olaparib or non-platinum–based chemotherapy, olaparib treatment resulted in a significantly greater objective response rate (ORR) and PFS [10]. Identification and selection of patients with PSROC who may derive the greatest magnitude of benefit from PARP inhibitors is therefore of particular interest.

Here, we describe results of the primary analysis of LIGHT (oLaparib In HRD-Grouped Tumor types; NCT02983799), a prospective study of treatment with olaparib monotherapy in patients with PSROC and known BRCAm and homologous recombination deficiency (HRD) status.

## 2. Methods

### 2.1. Study design and patients

LIGHT was a phase II, open-label, non-randomized, non-comparative, multicenter study in the USA and Canada. Eligible patients were ≥18 years old and had relapsed ovarian, primary peritoneal, and/or fallopian tube cancer that was histologically confirmed as high-grade serous or endometrioid with measurable disease (per the Response Evaluation Criteria in Solid Tumors [RECIST] version 1.1; ≥1 lesion that can be accurately assessed at baseline).

Following a protocol amendment (October 10, 2017) that reduced the number of prior lines of platinum-based chemotherapy from ≥2, eligible patients must have received ≥1 prior line of platinum-based chemotherapy. Eligible patients had disease progression ≥6 months after the end of their last platinum-based chemotherapy regimen. No prior PARP inhibitors were permitted. Complete eligibility criteria are described in the study protocol.

**Fig. 1.**
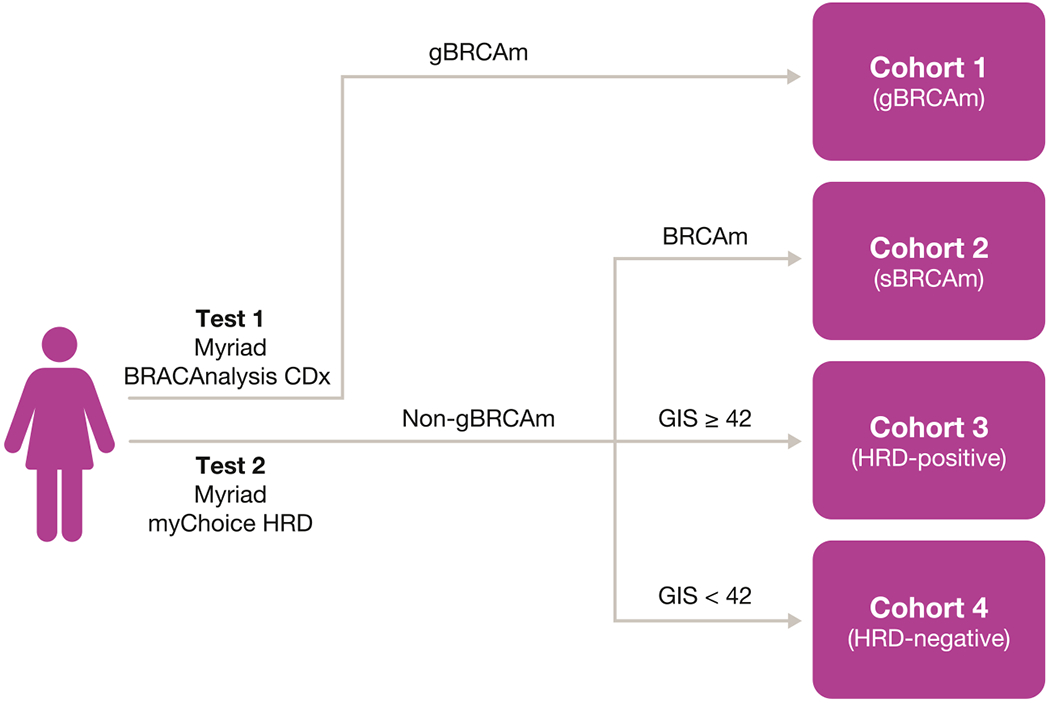
Patient assignment to cohorts in the LIGHT study. The Myriad myChoice^®^ HRD assay was FDA-approved as a companion diagnostic assay for olaparib in October 2019 and renamed myChoice CDx^®^. BRCAm, *BRCA1* and/or *BRCA2* mutation; FDA, US Food and Drug Administration; gBRCAm, germline BRCAm; GIS, genomic instability score; HRD, homologous recombination deficiency; LIGHT, oLaparib In Germline-, HRD-, and Tumor-mutated versus wild-type ovarian cancer; sBRCAm, somatic BRCA mutation.

Patients were assigned to cohorts according to BRCAm and HRD status based on the Myriad BRACAnalysis CDx^®^ and myChoice^®^ HRD assays (Cohort 1: presence of gBRCAm; Cohort 2: presence of somatic BRCAm [sBRCAm]; Cohort 3: HRD-positive [genomic instability score ≥42] without BRCAm; Cohort 4: HRD-negative [genomic instability score < 42]; Fig. 1). Patients received olaparib tablets 300 mg twice daily (BID) until investigator-assessed disease progression, unacceptable toxicity, or other protocol-specified criteria. Patients without a Myriad test result were not assigned to a cohort but were still permitted to receive study treatment.

The protocol was approved by ethics review committees at the participating institutions. The trial was performed in accordance with the Declaration of Helsinki, Good Clinical Practice guidelines, and the AstraZeneca policy on bioethics [11]. All patients provided written informed consent before enrollment.

### 2.2. Endpoints and assessments

The primary endpoint was investigator-assessed ORR (RECIST version 1.1). Secondary endpoints included disease control rate (DCR), investigator-assessed PFS, cancer antigen-125 response rate (CA-125 RR), time to any progression (TTAP), and safety.

Adverse events (AEs) were continually monitored from informed consent. Treatment-emergent AEs (TEAEs) were defined as new or worsening of prior AEs following the first dose of study treatment through 30 days after the last dose of study treatment. AEs were coded to preferred terms using the Medical Dictionary for Regulatory Activities (MedDRA^®^) version 22.0, with AE severity graded using the Common Terminology Criteria for Adverse Events (CTCAE) version 4.03. Anemia, neutropenia, and thrombocytopenia are presented as grouped terms that included multiple preferred terms (defined in the Supplementary Material). Additional information on AE management with dose modifications is in the study protocol.

Tumor assessments were conducted at baseline and every 8 weeks (±1 week) from receipt of first dose of olaparib for up to 48 weeks, and 12 weeks thereafter until investigator-assessed progression as determined by computed tomography or magnetic resonance imaging per RECIST v1.1 criteria (including confirmatory RECIST scan). Detailed information on study assessment timings is given in the study protocol.

### 2.3. Statistical analyses

The four cohorts were analyzed separately with no statistical comparison.

Based on precision estimates (95% CIs calculated using the Clopper–Pearson method), a target sample size of ≥30 patients for each cohort was identified to provide an adequate level of confidence in the estimated ORR. However, given the estimated number of patients expected in each genetic cohort, a maximum of 90 patients may have been enrolled in each of Cohorts 1, 3, and 4 by the time sufficient patients were enrolled in Cohort 2. With 30 patients, the maximum precision of the estimated ORR is approximately ±19%, and approximately ±11% with 90 patients.

The full analysis set included all patients enrolled. The efficacy analysis set included all patients who received ≥1 dose of olaparib and had a baseline tumor assessment indicating measurable disease. The safety analysis set included all patients who received ≥1 dose of olaparib.

ORR was defined as the proportion of patients with a best overall response of complete or partial response (CR/PR; assessed by investigators using RECIST version 1.1). CR or PR must have been confirmed ≥4 weeks after initial assessment. Responses were included until disease progression, last evaluable assessment in the absence of progression, or start of subsequent anti-cancer therapy.

DCR was defined as the proportion of patients with a best overall response of confirmed CR, PR, or stable disease (SD) for ≥8 weeks from the first dose.

CA-125 response was evaluated in patients with baseline CA-125 at least twice the upper limit of normal ≤2 weeks prior to treatment and was defined according to Gynecologic Cancer InterGroup (GCIG) criteria as a 50% reduction in CA-125 from baseline, confirmed and maintained for ≥28 days. CA-125 RR was the percentage of patients achieving a response. For CA-125 complete response, CA-125 must have additionally fallen within the normal range [12].

95% CIs for ORR, DCR, CA-125 RR, and CA-125 complete response rate in each cohort were calculated using the Clopper–Pearson method.

PFS was defined as the time from the first dose of olaparib until either disease progression (investigator assessed using RECIST version 1.1) or death. Patients without a PFS event at the data cut-off (DCO) were censored at the date of their latest tumor assessment.

TTAP was defined as the time from the first dose of olaparib until disease progression (RECIST version 1.1 or CA-125) or death.

PFS and TTAP in each cohort were analyzed using the Kaplan–Meier method, with Brookmeyer-Crowley 95% CIs for the medians.

The DCO (August 27, 2019) for the primary analysis presented here was approximately 6 months after the last patient was enrolled. An overall survival analysis is planned to be conducted 12 months after the primary analysis.

## 3. Results

### 3.1. Patients

From December 2016 to February 2019, 272 patients were enrolled and 259 of these were assigned to the 4 predefined cohorts (Cohort 1 [*n* = 75]; Cohort2 [*n* = 26]; Cohort3 [*n* = 68]; Cohort4 [*n* = 90]).Thirteen patients (4.8%) were unable to be assigned to a cohort as they had a failed or missing Myriad test result (one patient had missing gBRCAm status, and 12 had a failed and/or missing genomic instability score). In patients where a genomic instability score failed, reasons contributing to failure mainly included low tumor content in samples, no tumor in samples or low tumor DNA content detected in samples. Overall, 271 patients were included in the safety analysis set and 270 patients were included in the efficacy analysis set.

Baseline characteristics are shown in Table 1. Overall, patients had a median age at enrollment of 66 years, with a median time from primary diagnosis of 32.4 months. In total, 194 patients (71.3%) had International Federation of Gynecology and Obstetrics (FIGO) stage III disease at diagnosis and 181 (66.5%) had an Eastern Cooperative Oncology Group (ECOG) performance status of 0 at study entry.

At DCO, 207 patients (76.4%) had discontinued treatment, mostly due to disease progression (Cohort 1 [*n* = 43; 57.3%]; Cohort 2 [*n* = 15; 60.0%]; Cohort 3 [*n* = 52; 76.5%]; Cohort 4 [*n* = 86; 95.6%]; unassigned group [*n* = 11; 84.6%]). Of these, 58 patients (21.3%) had died.

### 3.2. Efficacy

**Fig. 2.**
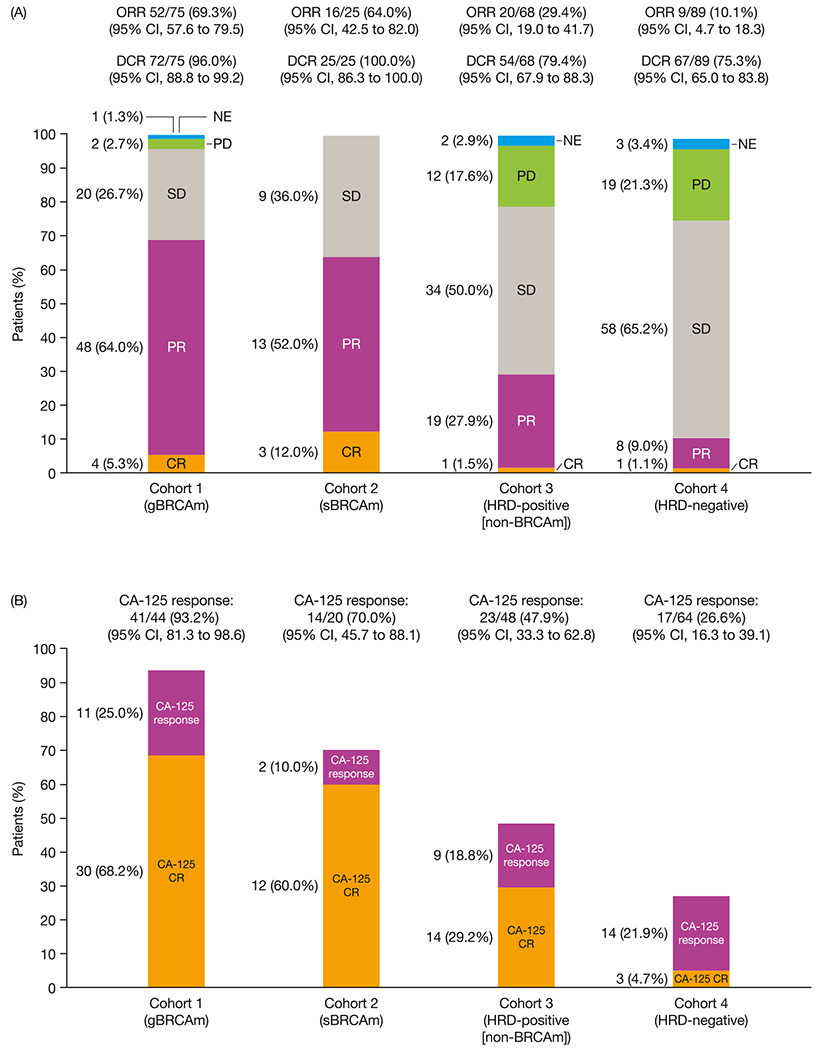
Best overall response by cohort (efficacy analysis set). BRCAm, *BRCA1* and/or *BRCA2* mutation; CI, confidence interval; CR, complete response; DCR, disease control rate; gBRCAm, germline BRCA mutation; HRD, homologous recombination deficiency; NE, not evaluable; ORR, objective response rate; PD, progressive disease; PR, partial response; sBRCAm, somatic BRCAm; SD, stable disease.

Objective responses were observed in all cohorts. The ORR (95% CI; *n*) was 69.3% (57.6–79.5; 52/75) in Cohort 1, 64.0% (42.5–82.0; 16/25) in Cohort 2, 29.4% (19.0–41.7; 20/68) in Cohort 3, 10.1% (4.7–18.3; 9/89) in Cohort 4 (Fig. 2), and 30.8% (9.1–61.4; 4/13) in the unassigned group. CA-125 responses and CRs were observed among 176 evaluable patients across the cohorts; the highest CA-125 RRs and CRs were in the BRCAm cohorts. Among patients without a BRCAm, higher CA-125 responses were seen in the HRD-positive cohort (Supplementary Fig. S1).

**Fig. 3.**
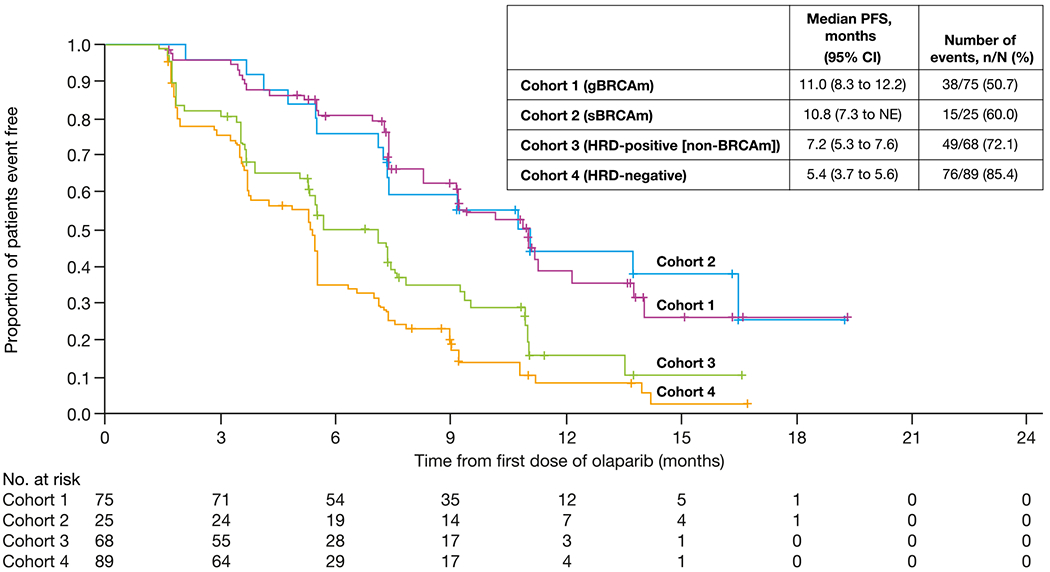
Kaplan–Meier plot of PFS by cohort (efficacy analysis set). BRCAm, *BRCA1* and/or *BRCA2* mutation; gBRCAm, germline BRCAm; CI, confidence interval; HRD, homologous recombination deficiency; PFS, progression-free survival; NE, not estimable; sBRCAm, somatic BRCAm.

At DCO, PFS events had been observed in 50.7% (*n* = 38/75), 60.0% (*n* = 15/25), 72.1% (*n* = 49/68), 85.4% (*n* = 76/89), and 69.2% (*n* = 9/13) in Cohorts 1, 2, 3, 4, and the unassigned group, respectively. Median PFS (95% CI) was 11.0 (8.3–12.2), 10.8 (7.3 to not estimable [NE]), 7.2 (5.3–7.6), 5.4 (3.7–5.6) in each cohort, respectively (Fig. 3), and was 9.2 (3.5–12.7) months in the unassigned group. Six-month PFS rates were 80.8%, 76.0%, 50.3%, and 34.9% in each cohort, and 55.0% in the unassigned group, respectively. The median (range) duration of follow-up among patients censored for PFS was 9.2 (1.7–19.4) months in Cohort 1, 12.4 (7.4–19.3) months in Cohort 2, 7.4 (0.0–16.6) months in Cohort 3, 8.8 (0.0–16.8) months in Cohort 4, and 6.2 (0.0–13.7) months in the unassigned group.

ORR and PFS were generally consistent within cohorts irrespective of the number of prior lines of chemotherapy subgroups (1 vs ≥ 2) (Supplementary Fig. S2, Supplementary Fig. S3).

Median TTAP was longest in the BRCAm cohorts, consistent with other efficacy endpoints; among patients without a BRCAm, median TTAP was longest in HRD-positive patients (Supplementary Fig. S4).

### 3.3. Safety

In the safety analysis set, the median (range) total duration of treatment was 7.3 (0.5–22.2) months and the median actual duration of treatment (excluding dose interruptions) was 7.1 (0.1–21.9) months.

Overall, TEAEs and CTCAE Grade ≥ 3 TEAEs were reported in 98.5% and 43.5% of patients, respectively; TEAEs reported in ≥15% of patients are shown in Table 2. The majority of TEAEs were CTCAE Grade 1–2; the most common CTCAE Grade ≥ 3 TEAE was anemia (15.1%) (Table 2). The 4 most common TEAEs (nausea, fatigue/asthenia, vomiting, and anemia) typically occurred early (median time to first occurrence 5–52 days; Supplementary Table S1). Over the first 12 months of treatment, both the prevalence and severity of nausea and vomiting decreased. While the prevalence of fatigue/asthenia and anemia plateaued early, their overall severity decreased over time (Supplementary Fig. S5). Treatment-related (as assessed by the investigator) serious TEAEs were reported in 20 patients (7.4%), including anemia in 3 patients (1.1%); no other treatment-related serious TEAEs occurred in >2 patients.

Neutropenia and thrombocytopenia were reported in 19 patients (7.0%) and 14 patients (5.2%), respectively, including 6 patients (2.2%) and 3 patients (1.1%) who experienced CTCAE Grade ≥ 3 neutropenia (1 CTCAE Grade 4) and thrombocytopenia (none CTCAE Grade 4), respectively. Among AEs of special interest, 1 patient (0.4%) each experienced pneumonitis (CTCAE Grade 3) and pulmonary fibrosis (CTCAE Grade 2; Supplementary material). No events of acute myeloid leukemia (AML), myelodysplastic syndrome (MDS) or new primary malignancies were reported.

Most patients (150 [55.4%]) did not require dose modification (including interruption and reduction) from the planned starting dose of 300 mg BID. Most dose reductions occurred early in treatment. At 12 months, 46/66 patients (69.7%) remained on the starting dose of olaparib (Supplementary Fig. S6). TEAEs leading to dose interruptions and reductions were reported in 90 patients (33.2%) and 66 (24.4%), respectively; including 26 (9.6%) who required both a dose interruption and reduction. TEAEs leading to olaparib discontinuation were reported in 12 patients (4.4%; Supplementary Table S2). No single TEAE led to discontinuation in >2 patients. There was one TEAE with a fatal outcome (atrial fibrillation 46 days after the start of olaparib in a patient with a history of atrial fibrillation); the TEAE was considered unrelated to olaparib by the investigator.

## 4. Discussion

To our knowledge, LIGHT is the first prospective study to evaluate olaparib treatment in patients with PSROC who had received ≥1 prior line of platinum-based chemotherapy and who were assigned to cohorts according to known BRCAm and HRD status. Response to olaparib was seen across the cohorts and patients with a BRCAm had the highest ORRs (69% and 64% in the gBRCAm and sBRCAm cohorts, respectively) and longest PFS (~11 months). Similar efficacy was observed in both the gBRCAm and sBRCAm cohorts, consistent with prior reports in the PSROC setting [13–15]. Among patients without a BRCAm, greater efficacy was seen in those with a HRD-positive tumor, with an ORR of 29% and a median PFS of 7 months. Efficacy was generally similar within cohorts irrespective of the number of prior lines of chemotherapy.

Previous reports have demonstrated the efficacy of PARP inhibition for the treatment of PSROC outside of the maintenance setting. The SOLO3 study reported a significant improvement in ORR as assessed by blinded independent central review (72.2% vs 51.4%; odds ratio [OR] 2.53 [95% CI 1.40–4.58]) and investigator-assessed PFS (HR 0.62 [95% CI, 0.43–0.91]) with olaparib treatment over non-platinum chemotherapy in patients with PSROC and a BRCAm who had received ≥2 prior lines of platinum-based chemotherapy [10]. Similarly, among patients with a gBRCAm in the phase III GY004 study of olaparib monotherapy or olaparib plus cediranib versus platinum-based chemotherapy as treatment for PSROC, the HR for PFS with olaparib monotherapy versus platinum-based chemotherapy was 0.63 (95% CI 0.37–1.07).

Other trials, with niraparib [16,17] and rucaparib [14], have demonstrated the efficacy of PARP inhibitors as treatment in populations beyond patients with a BRCAm. In the phase II AVANOVA2 study of niraparib with/without bevacizumab in PSROC, the niraparib monotherapy arm (including 37% with a BRCAm and 55% with one prior line of platinum-based chemotherapy) had an ORR of 27% (95% CI, 15–41) and median PFS of 5.5 months [16]. In the phase II QUADRA study of patients with PSROC (48% of patients had HRD-positive tumors), 13 of 47 patients (28%; 95% CI, 16–43) who had received ≥3 prior lines of therapy achieved a confirmed objective response to niraparib monotherapy with a median PFS of 5.5 months [17]. In part one of the phase II ARiEL2 study of rucaparib monotherapy, also in PSROC, 32 of 40 patients (80%; 95% CI, 64–91) achieved an objective response among patients with a BRCAm, 24 of 82 patients (29%; 95% CI, 20–40) without a BRCAm and with high loss of heterozygosity (LOH), and 7 of 70 patients (10%; 95% CI, 4–20) without a BRCAm and low LOH [14]. Although any cross-trial comparisons should only be made with caution due to disparate patient populations and procedures (particularly for assessing HRD status), it is notable in each of the previously mentioned studies [14,16,17] that the highest ORRs and longest PFS with PARP inhibitor treatment were observed in patients with BRCAm, HRD-positive tumors, or high LOH, consistent with the findings in LIGHT. For patients without a BRCAm in this study, a higher ORR and longer median PFS and TTAP were observed in the HRD-positive cohort than in the HRD-negative cohort. These data are consistent with the established utility of HRD (by BRCAm and/or genomic instability) as an indicator of particular benefit from PARP inhibition [14,15,18–21].

The observed RECIST and CA-125 responses even in the HRD-negative cohort also suggest that platinum sensitivity is a key factor to predict response to PARP inhibitor treatment in PSROC. In addition, the high DCR of >75% across all four cohorts suggests that a wide range of patients with PSROC may derive clinical benefit from PARP inhibitor treatment, consistent with other studies [14,15,18–20]. Therefore, olaparib may represent an effective chemotherapy-sparing treatment for all patients with PSROC, although the magnitude of benefit is greater in patients with BRCAm and/or HRD-positive tumors.

Multiple tests have been used to identify patients with PSROC who may particularly benefit from PARP inhibitor therapy. This study used the Myriad myChoice^®^ HRD assay, with a genomic instability score of ≥42 deemed HRD-positive. This assay is based on LOH, telomeric allelic imbalance, and large-scale state transitions and is consistent with testing methodology used in the AVANOVA2 and QUADRA studies [16,17]. However, in ARIEL2, subgroups were defined using BRCAm and LOH, as determined by Foundation Medicine T5 testing [14]. Refinement of the markers used to select patients who may benefit would inform optimal treatment selection in PSROC. The ORR of approximately 30% in the HRD-positive (non-BRCAm) cohort in this study and in similar populations in other studies suggests that HRD assays may identify a patient population who can benefit from PARP inhibition. However, the higher ORRs in the BRCAm cohorts (69% and 64% for gBRCAm and sBRCAm, respectively) suggest that BRCAm is the best biomarker so far to predict response to PARP inhibition.

Olaparib treatment was well tolerated and the safety profile was consistent with that seen in prior olaparib studies [10,22]. The median total duration of treatment was similar to the median actual duration of treatment (ie, excluding dose interruptions), suggesting dose interruptions did not have a notable impact. The majority of TEAEs with olaparib treatment were manageable with dose modification and supportive treatment and there was a low discontinuation rate due to TEAEs.

Limitations of this study include its non-randomized nature and lack of a comparator arm. The protocol amendment that reduced the required number of prior lines of platinum-based chemotherapy from ≥2 to ≥1 meant that there were proportionally fewer patients who had received one line of therapy in the HRD-negative cohort, which was the fastest to enroll. The evolving ovarian cancer therapeutic landscape, with PARP inhibitor efficacy demonstrated as first-line maintenance (with or without bevacizumab), increases the likelihood of prior PARP inhibitor exposure among patients who receive PARP inhibition for PSROC. Although analyses have suggested some patients may derive benefit from PARP inhibitor retreatment [23,24], and the phase III OReO trial reported a PFS benefit with maintenance olaparib rechallenge [25], data fully exploring PARP inhibition in the treatment setting following prior PARP inhibitor exposure are limited.

In conclusion, as previously observed in trials of PARP inhibitors in and outside of the maintenance setting, olaparib treatment for patients with PSROC in LIGHT demonstrated greatest efficacy among patients with a BRCAm, with similar efficacy seen with both gBRCAm and sBRCAm. For patients without a BRCAm, a higher ORR and longer median PFS were observed in the HRD-positive cohort than in the HRD-negative cohort. Therefore, olaparib may represent an effective chemotherapy-sparing treatment for all patients with PSROC.

## Supplementary Material

1

## Figures and Tables

**Table 1:** Baseline characteristics by cohort (full analysis set).

	Cohort 1 (gBRCAm) (*n* = 75)	Cohort 2 (sBRCAm) (*n* = 26)	Cohort 3 (HRD-positive non-BRCAm) (*n* = 68)	Cohort 4 (HRD-negative) (*n* = 90)	Unassigned^a^ (*n* = 13)	Overall (*N* = 272)
Median age, years (range)	61 (42–80)	71 (50–90)	64 (38–88)	69 (35–91)	72 (45–81)	66 (35–91)
Median time from primary diagnosis, months	26.1	31.7	35.4	34.5	20.9	32.4
Median prior lines of chemotherapy, *n* (range)	1.0 (1–4)	2.0 (1–5)	2.0 (1–4)	2.0 (1–7)	1.0 (1–5)	2.0 (1–7)
*Histology*						
Serous	68 (90.7)	24 (92.3)	65 (95.6)	83 (92.2)	13 (100.0)	253 (93.0)
Other	7 (9.3)	2 (7.7)	3 (4.4)	7 (7.8)	0	19 (7.0)
*Tumor grade*						
High grade	51 (68.0)	21 (80.8)	53 (77.9)	67 (74.4)	13 (100.0)	205 (75.4)
Poorly differentiated (G3)	21 (28.0)	5 (19.2)	13 (19.1)	18 (20.0)	0	57 (21.0)
Moderately differentiated (G2)	3 (4.0)	0	1 (1.5)	4 (4.4)	0	8 (2.9)
Well differentiated (G1)	0	0	1 (1.5)	0	0	1 (0.4)
Missing	0	0	0	1 (1.1)	0	1 (0.4)
*FIGO stage at original diagnosis*						
I	5 (6.7)	3 (11.5)	0	4 (4.4)	0	12 (4.4)
II	5 (6.7)	1 (3.8)	4 (5.9)	5 (5.6)	0	15 (5.5)
III	53 (70.7)	14 (53.8)	53 (77.9)	65 (72.2)	9 (69.2)	194 (71.3)
IV	12 (16.0)	8 (30.8)	11 (16.2)	16 (17.8)	3 (23.1)	50 (18.4)
Missing	0	0	0	0	1 (7.7)	1 (0.4)
*ECOG performance status*						
0	58 (77.3)	13 (50.0)	48 (70.6)	56 (62.2)	6 (46.2)	181 (66.5)
1	17 (22.7)	12 (46.2)	20 (29.4)	34 (37.8)	7 (53.8)	90 (33.1)
Missing	0	1 (3.8)	0	0	0	1 (0.4)

NOTE. Data are *n* (%) unless otherwise stated.

BRCAm, *BRCA1* and/or *BRCA2* mutation; ECOG, Eastern Cooperative Oncology Group; FIGO, International Federation of Gynecology and Obstetrics; gBRCAm, germline BRCAm; HRD, homologous recombination deficiency; sBRCAm, somatic BRCAm.

a Includes patients who were not assigned to a cohort as they had a Myriad test result of failed or missing.

**Table 2.** TEAEs (safety analysis set).

	Any CTCAE grade	CTCAE Grade ≥ 3
	(*N* = 271)	(N = 271)
	*n* (%)	*n* (%)
*Any TEAE*	267 (98.5)	118 (43.5)
Treatment-related TEAE	252 (93.0)	71 (26.2)
Serious TEAE	67 (24.7)	NA
Treatment-related serious TEAE	20 (7.4)	NA
TEAE leading to discontinuation	12 (4.4)	NA
TEAE leading to dose reduction	66 (24.4)	NA
TEAE leading to dose interruption	90 (33.2)	NA
*Most common TEAEs (≥15%)*		
Nausea	180 (66.4)	5 (1.8)
Fatigue/asthenia^a^	168 (62.0)	11 (4.1)
Vomiting	89 (32.8)	3 (1.1)
Anemia^a^	78 (28.8)	41 (15.1)
Constipation	66 (24.4)	1 (0.4)
Diarrhea	60 (22.1)	3 (1.1)
Decreased appetite	59 (21.8)	1 (0.4)
Abdominal pain	52 (19.2)	5 (1.8)
Headache	52 (19.2)	1 (0.4)
Increased blood creatinine	45 (16.6)	0
Cough	44 (16.2)	1 (0.4)
Dyspnea	44 (16.2)	5 (1.8)
Dizziness	42 (15.5)	1 (0.4)
Dysgeusia	41 (15.1)	0

CTCAE, Common Terminology Criteria for Adverse Events; NA, not available; TEAE, treatment-emergent adverse event.

a Grouped term.

## Data Availability

Data underlying the findings described in this manuscript may be obtained in accordance with AstraZeneca’s data-sharing policy described at https://astrazenecagrouptrials.pharmacm.com/ST/Submission/Disclosure.
