# Supplementary material for: Olaparib treatment for platinum-sensitive relapsed ovarian cancer by BRCA mutation and homologous recombination deficiency status Phase II LIGHT study primary analysis
Source: Gynecol Oncol. Author manuscript; Available in PMC 2023 Feb 9. (PMC9909678; doi:10.1016/j.ygyno.2022.06.017)
Supplement: 1 [file NIHMS1868098-supplement-1.docx]

# Supplementary material to:

# Olaparib Treatment for Platinum-Sensitive Relapsed Ovarian Cancer by BRCA Mutation and Homologous Recombination Deficiency Status: Phase II LIGHT Study Primary Analysis

Karen Cadoo, Fiona Simpkins, Cara Mathews, Ying L. Liu, Diane Provencher, Colleen McCormick, Adam C. ElNaggar, Alon D. Altman, Lucy Gilbert, Destin Black, Nashwa Kabil, James Bennett, Jiefen Munley, Carol Aghajanian

## Supplementary methods

## Myriad testing methodology

Presence of germline BRCAm (gBRCAm) was determined using the BRACAnalysis CDx^®^ test (Myriad Genetic Laboratories, Inc., Salt Lake City, UT, USA). Homologous recombination deficiency (HRD; using a genomic instability score (GIS) cutoff of ≥ 42) and somatic BRCAm (sBRCAm) statuses were determined using the myChoice^®^ HRD test (Myriad Genetic Laboratories, Inc.), which tests for tumor BRCAm (tBRCAm) and determines the GIS based on loss of heterozygosity (LOH), telomeric allelic imbalance, and large-scale state transitions. Patients with a negative BRACAnalysis CDx^®^ test but a positive myChoice^®^ HRD BRCAm result were classified as having a sBRCAm.

## Definitions of grouped treatment-emergent adverse events (MedDRA preferred terms)

Anemia was a grouped term that included queries for the Medical Dictionary for Regulatory Activities (MedDRA^®^) preferred terms of anaemia, anaemia macrocytic, erythropenia, haematocrit decreased, haemoglobin decreased, normochromic anaemia, normochromic normocytic anaemia, normocytic anaemia, and red blood cell count decreased.

Neutropenia was a grouped term that included queries for the MedDRA preferred terms of agranulocytosis, febrile neutropenia, granulocyte count decreased, granulocytopenia, idiopathic neutropenia, neutropenia, neutropenic infection, neutropenic sepsis, and neutrophil count decreased.

Thrombocytopenia was a grouped term that included queries for the MedDRA preferred terms of platelet count decreased, platelet production decreased, plateletcrit decreased, and thrombocytopenia.

## Supplementary results

## Adverse events of special interest

There was one confirmed case of CTCAE Grade 2 pneumonitis 57 days after the start of olaparib treatment; the event resolved after treatment discontinuation. The same patient subsequently experienced CTCAE Grade 3 pneumonitis that began 108 days after the last dose of olaparib. In another patient, CTCAE Grade 2 pneumonitis that began 22 days after the last dose of olaparib was reported; however, this was later reclassified as pneumonia by the investigator. In both cases, the events were considered by the investigator to be causally related to olaparib treatment.

One patient experienced CTCAE Grade 2 pulmonary fibrosis that began 110 days after the start of olaparib treatment and was considered by the investigator to be causally related to olaparib treatment.

## Table S1. Time to and duration of first event for common treatment-emergent adverse events (safety analysis set)

|  | **Median time to first occurrence, days  (Q1–Q3)** | **Median duration of first event,^†^ days** |
| --- | --- | --- |
| Nausea | 5 (2–12) | 57 |
| Fatigue/asthenia* | 15 (5–47) | NR |
| Vomiting | 21 (7–99) | 2 |
| Anemia* | 52 (15–57) | 305 |

NR, not reached.

*Grouped term.

^†^Estimated using Kaplan–Meier method (patients with no recorded adverse event end date were censored at the date of data cut-off or the date withdrawn from the study, if earlier).

## Table S2. Treatment-emergent adverse events leading to discontinuation of olaparib (safety analysis set)

|  | **Overall**  **(N=271)**  **n (%)** |
| --- | --- |
| Any | 12 (4.4) |
| CTCAE Grade 3 | 7 (2.6) |
| CTCAE Grade 2 | 3 (1.1) |
| CTCAE Grade 1 | 2 (0.7) |
| Fatigue/asthenia*^†^ | 2 (0.7) |
| Nausea^‡^ | 2 (0.7) |
| Chronic kidney disease | 1 (0.4) |
| Cough^†^ | 1 (0.4) |
| Dizziness | 1 (0.4) |
| Drug hypersensitivity | 1 (0.4) |
| Gastrointestinal obstruction | 1 (0.4) |
| Intestinal obstruction | 1 (0.4) |
| Memory impairment | 1 (0.4) |
| Pneumonitis | 1 (0.4) |
| Small intestinal obstruction | 1 (0.4) |
| Vomiting^‡^ | 1 (0.4) |

CTCAE, Common Terminology Criteria for Adverse Events.

*Grouped term; ^†^One patient discontinued olaparib due to cough and fatigue/asthenia; ^‡^One patient discontinued olaparib due to nausea and vomiting.

## Figure S1. CA-125 response and CR rate by cohort (efficacy analysis set)


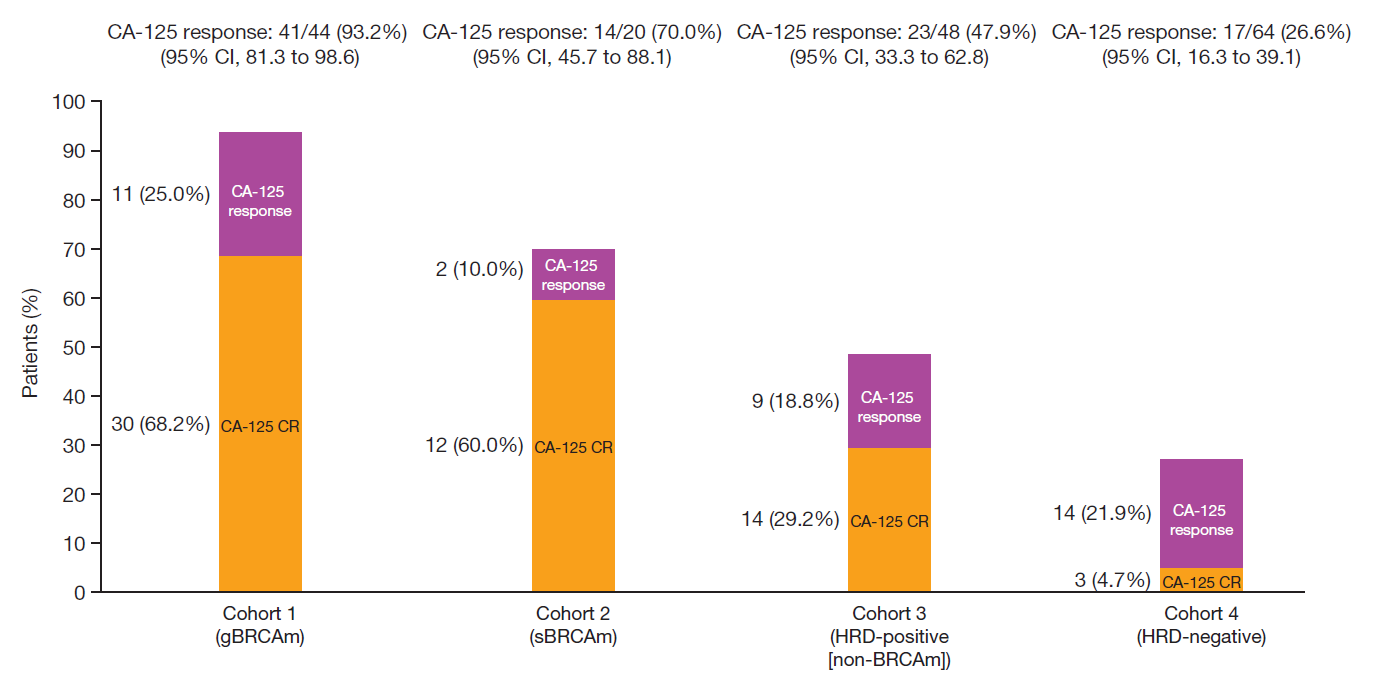


BRCAm, *BRCA1* and/or *BRCA2* mutation; CA-125, cancer antigen-125; CI, confidence interval; CR, complete response; gBRCAm, germline BRCAm; HRD, homologous recombination deficiency; NE, not evaluable; sBRCAm, somatic BRCAm.

**Figure S2. ORR by number of prior lines of chemotherapy (efficacy analysis set)**


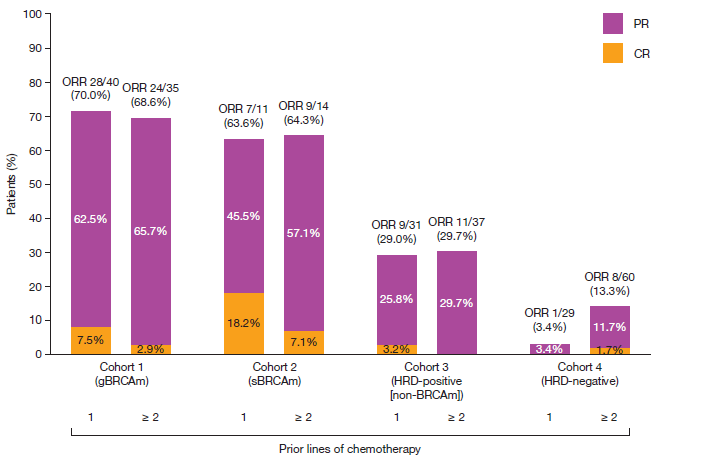


BRCAm, *BRCA1* and/or *BRCA2* mutation; CR, complete response; gBRCAm, germline BRCAm; HRD, homologous recombination repair; ORR, objective response rate; PR, partial response; sBRCAm, somatic BRCAm.

## Figure S3. Kaplan–Meier plot of PFS by number of prior lines of chemotherapy (efficacy analysis set)

### A


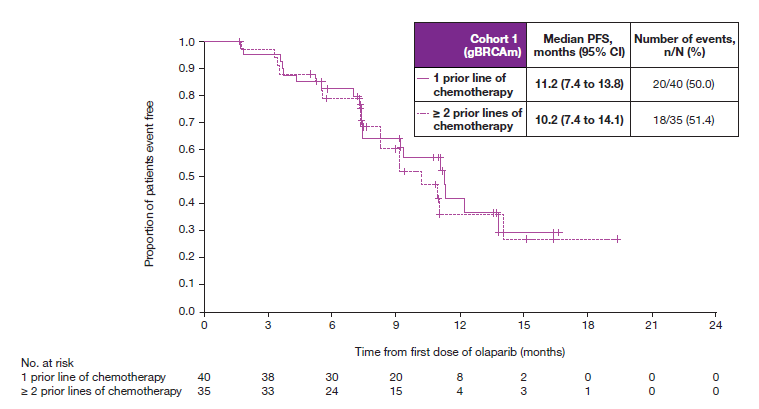


### B


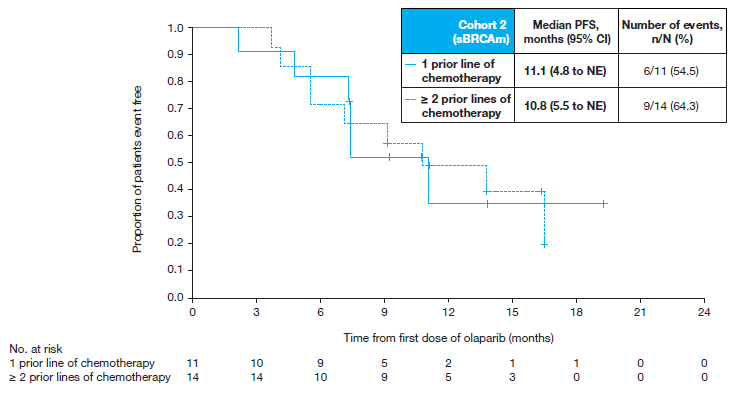


### C


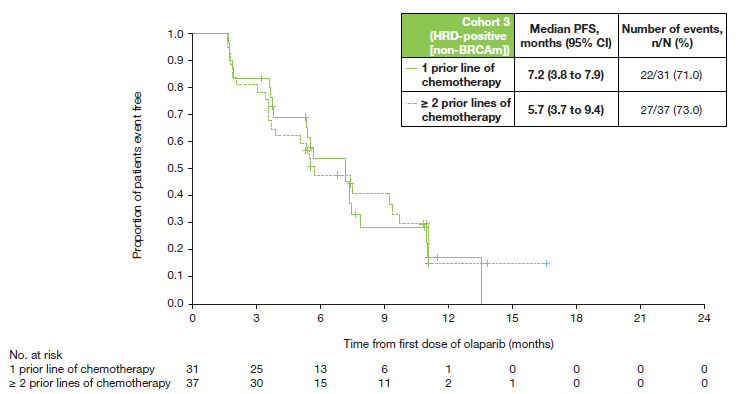


### D


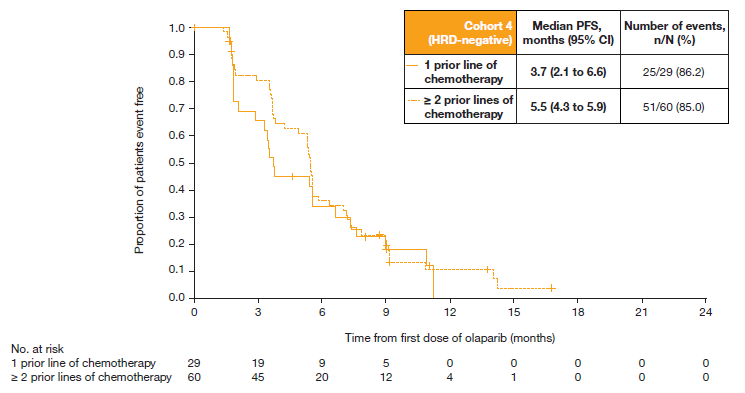


BRCAm, *BRCA1* and/or *BRCA2* mutation; CI, confidence interval; gBRCAm, germline BRCAm; HRD, homologous recombination repair; PFS, progression-free survival; sBRCAm, somatic BRCAm.

## Figure S4. Kaplan–Meier plot of TTAP (efficacy analysis set)


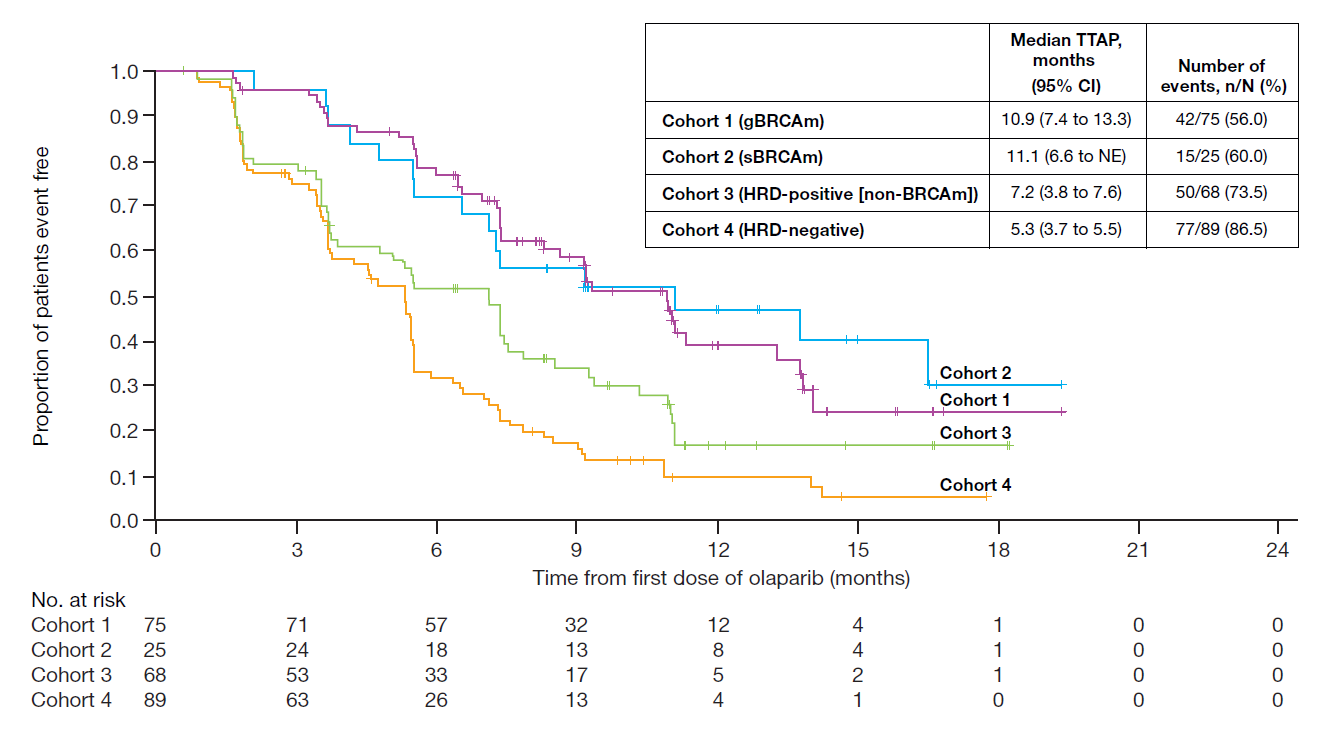


BRCAm, *BRCA1* and/or *BRCA2* mutation; CI, confidence interval; gBRCAm, germline BRCAm; HRD, homologous recombination deficiency; NE, not estimable; sBRCAm, somatic BRCAm; TTAP, time to any progression.

## Figure S5. Prevalence and severity of the 4 most common treatment-emergent adverse events over the first 12 months of olaparib treatment (safety analysis set)


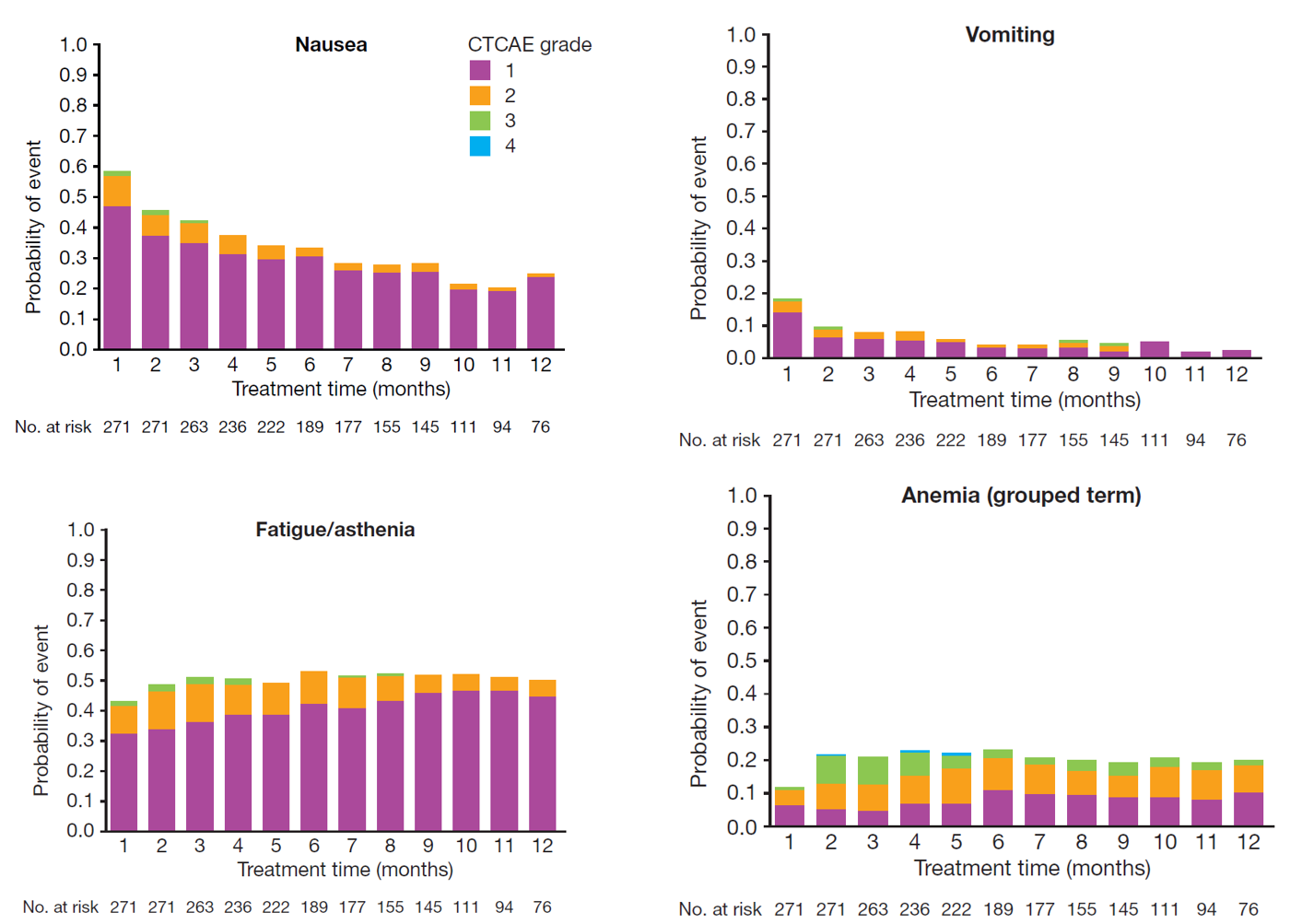


The highest-grade toxicity adverse event in each month is considered per patient. Patients are counted once per month. Number at risk is based on the number at risk at the beginning of the interval.

CTCAE, Common Terminology Criteria for Adverse Events.

## Figure S6. Dosing changes over the first 12 months of olaparib treatment (safety analysis set)

##
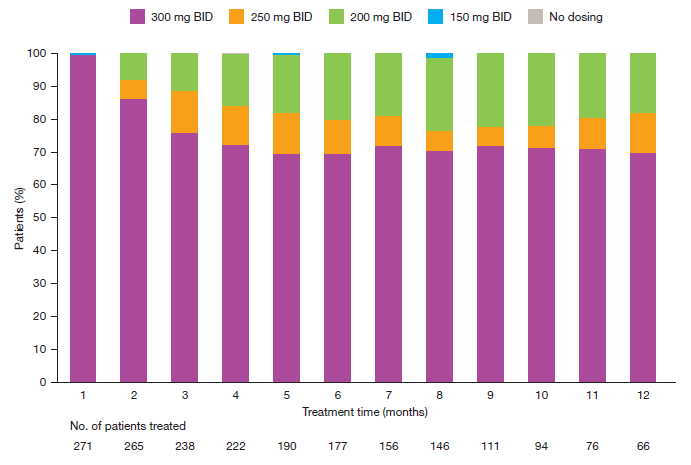


Each patient was counted at the first non-missing dose level within the given month window. If a patient had dosing interrupted for the entire month window, the category of ‘No dosing’ was assigned.

BID, twice daily.
